# Pediatric nephrotic syndrome: The interplay of oxidative stress and inflammation

**DOI:** 10.5937/jomb0-46526

**Published:** 2024-06-15

**Authors:** Simachew Yonas Mulat, Marija Mihajlović, Tamara Antonić, Gordana Miloševski-Lomić, Amira Peco-Antić, Dragana Jovanović, Dušan Paripović, Aleksandra Stefanović

**Affiliations:** 1 University of Belgrade, Faculty of Pharmacy, Department of Medical Biochemistry, Belgrade; 2 University Children's Hospital, Department of Nephrology, Belgrade; 3 University of Belgrade, School of Medicine, Belgrade; 4 Internal Medicine Clinic "Akta Medica" , Belgrade

**Keywords:** nephrotic syndrome, oxidative stress, inflammation, hypertension, pentraxin 3, advanced oxidation protein products, nefrotski sindrom, oksidativni stres, upala, hipertenzija, pentrakin 3, napredni oksidacioni proteinski proizvodi

## Abstract

**Background:**

The pathophysiological mechanisms crucial in the development of nephrotic syndrome (NS) in the pediatric population are still not fully understood. This study aimed to investigate the relationship between hypertension, oxidative stress, and inflammation in pediatric patients during the acute phase of the disease.

**Methods:**

The study included 33 children, aged 2 to 9 years, with nephrotic syndrome. Blood samples were collected during the acute phase and remission. Parameters of oxidative status were determined, including total oxidative status (TOS), advanced oxidation protein products (AOPP), prooxidant-antioxidant balance (PAB), sulfhydryl groups (-SH), paraoxonase 1 (PON1), and total antioxidant status (TAS) in serum, measured spectrophotometrically. Inflam - matory parameters such as pentraxin 3 (PTX3), leptin, program med cell death ligand 1 (PD-L1), and E-cadherin were determined using enzyme-linked immunosorbent assay (ELISA).

**Results:**

Patients with nephrotic syndrome and hypertension had significantly higher levels of advanced oxidation protein products and total antioxidant status (p=0.029 and p=0.003, respectively). During the acute phase of the disease, lower activity of sulfhydryl groups and paraoxonase 1 was observed compared to remission (p<0.001, for both). Pentraxin 3 levels were higher, while leptin levels were lower during the acute phase (p<0.001, for both). Pentraxin 3 correlated with advanced oxidation protein products and total antioxidant status during the acute phase but not in remission (rs=0.42, p=0.027 and rs=0.43, p=0.025, respectively). A negative correlation between Advanced oxidation protein products and leptin was observed during the acute phase, which disappeared in remission (rs=-0.42, p=0.028).

**Conclusions:**

Results of this study show that hypertension influences oxidative stress markers, and decreased antioxidant capacity may contribute to nephrotic syndrome development. Pentraxin 3 appears as a potential disease activity marker, indicating a dynamic connection between inflammation and oxidative stress. Leptin may also play a role in oxidative stress in nephrotic syndrome.

## Abstract (sr)

**Uvod:**

Patofiziološki mehanizmi ključni u razvoju nefrotskog sindroma (NS) u pedijatrijskoj populaciji još uvek nisu u potpunosti razjašnjeni. Ova studija ima za cilj proučavanje sinergističkog delovanja oksidativnog stresa i inflamacije u patogenezi NS. Takođe, jedan od ciljeva ove studije je i ispitivanje veze hipertenzije sa stepenom oksidativnog stresa i inflama - cije kod pacijenata u akutnoj fazi bolesti.

**Metode:**

U studiju je uključeno 33 dece sa NS uzrasta od 2 do 9 godina. Uzorci krvi su prikupljeni tokom akutne faze i remisije. Od parametara oksidativnog statusa određivani su: totalni oksidativni status (TOS), uznapredovali proizvodi oksidacije proteina (AOPP), balans prooksidans-antioksidans (PAB), sulfhidrilne grupe (-SH), paraoksonaza 1 (PON1) i ukupan antioksidativni status (TAS) u serumu su mereni spektrofometrijski, a od parametara inflamacije su pentraksin 3 (PTX3), leptin, ligand programirane smrti ćelije 1 (PD-L1) i E-kadherin određivani metodom enzimskog imunosorbentnog testa (ELISA).

**Rezultati:**

Pacijenti sa NS i hipertenzijom imali su značajno više nivoe AOPP i TOS (p=0.029 i p=0.003, respektivno). U akutnoj fazi bolesti su uočene nižu aktivnost -SH i PON1 u poređenju sa remisijom (p<0.001, za oba). Nivoi PTX 3 su bili viši, dok su nivoi leptina bili niži tokom akutne faze (p<0.001, za oba). PTX 3 je korelirao sa AOPP i TAS u akutnoj fazi, ali ne i u remisiji (rs=0.42, p=0.027 i rs=0.43, p=0.025,respektivno). U akutnooj fazi utvrđena je negativna korelacija između AOPP i leptina, koja je nestala u remisiji (rs=-0.42, p=0.028).

**Zaključak:**

Rezultati ove studije ukazuju da hipertenzija utiče na markere oksidativnog stresa, a smanjeni antioksidativni kapacitet može doprineti razvoju NS. PTX3 se pojavljuje kao potencijalni marker aktivnosti bolesti, što ukazuje na dinamičku vezu između inflamacije i oksidativnog stresa. Leptin može igrati ulogu u oksidativnom stresu u NS.

## Introduction

Nephrotic syndrome (NS) is a clinical condition that causes heavy proteinuria, hypoalbuminemia, hyperlipidemia, and edema due to an increased permeability of the glomerular filtration barrier [1]. It primarily affects the pediatric population with a reported incidence of 2–7 cases per 100,000 children [1]. While the cause of NS in pediatric patients is often idiopathic, inflammation and oxidative stress have been implicated in its pathogenesis and complications [1] [2]. Glucocorticoids are the primary treatment for idiopathic NS, leading to clinical remission in the majority of patients within 4 weeks.

Children with NS, irrespective of remission status or glucocorticoid exposure duration, display a higher prevalence of cardiovascular risk factors than the general pediatric population [3]. Hypertension is one of the common complications of NS and a well-established risk factor for kidney and cardiovascular diseases [3] [4]. The origin of hypertension in NS is multifactorial, encompassing sodium retention, impaired kidney function, albuminuria, genetic predisposition, and medication side effects [4]. Although inflammation and oxidative stress are linked to hypertension and its related conditions [5] [6], the particular influence of hypertension on inflammation and oxidative stress in NS patients, despite the high prevalence of hypertension in this population, has not been examined. Understanding these connections could identify novel therapeutic targets and inform tailored interventions to mitigate the adverse effects of hypertension in NS patients.

Oxidative stress denotes the state of imbalance between reactive oxygen species (ROS) and the antioxidant system that eliminates them, resulting in compromised cellular signaling and cell damage [7]. Oxidative stress can contribute to the development and progression of NS by damaging the glomerular filtration barrier, promoting inflammation, altering lipid metabolism, impairing endothelial function and causing cellular damage [2] [8]. Several studies have shown that NS patients experience high levels of oxidative stress during the acute phase of the condition, and some studies have reported that oxidative stress markers remain elevated even after steroidinduced remission of the disease [7] [9] [10]. Oxidative stress assessment entails evaluating both damaged stable molecules and antioxidant molecules, including enzymatic and non-enzymatic antioxidants. While earlier studies predominantly focused on lipid peroxidation in NS, notably malondialdehyde (MDA), protein oxidative damage in NS patients has been less explored. Advanced oxidation protein products (AOPP) are significant byproducts of oxidative stress in plasma proteins, activating the Wnt/β-catenin signaling pathway in glomerular podocytes and contributing to podocyte dysfunction and proteinuria [11]. Paraoxonase 1 (PON 1) is an antioxidant enzyme associated with high-density lipoprotein (HDL), and multiple studies have established a connection between reduced PON 1 activity and increased atherosclerosis risk [12]. Furthermore, protein sulfhydryl groups (-SH) have a pivotal role in the antioxidant defense system, with low serum -SH content associated with increased chronic kidney disease (CKD) risk [13]. Previous NS studies primarily measured individual pro-oxidant and anti-oxidant markers, despite their resource-intensive and time-consuming nature and limitations in capturing the combined prooxidant and antioxidant impact. Total oxidant status (TOS), total antioxidant status (TAS), and prooxidant- antioxidant balance (PAB) represent established parameters for assessing oxidative stress, with PAB underexplored in pediatric NS patients [14] [15] [16]. Such comprehensive parameters are particularly valuable in pediatric settings, where limited specimen quantities are prevalent.

In parallel, inflammation significantly contributes to the development and progression of NS [17] [18]. The intricate interplay between immune dysregulation and kidney dysfunction prompts investigations into various molecular players that contribute to this inflammatory milieu. Pentraxin 3 (PTX3), an acute-phase protein regulating innate immunity, inflammation, and tissue remodeling, has been associated with kidney dysfunction and disease severity in inflammatory, cardiovascular, and autoimmune conditions [19]. Moreover, PTX3 has been shown to predict the risk of developing CKD and cardiovascular events in patients with kidney diseases [19] [20]. PTX3's impact varies based on clinical circumstances, but its role in NS remains unexplored [21]. Leptin, an adipocyte-derived hormone, is also believed to play a role in many inflammation-related diseases, including cardiovascular and chronic kidney diseases [22]. In physiological conditions, leptin regulates energy homeostasis, glucose metabolism, lipid metabolism, and immune function [22]. Several studies have shown that a high level of leptin concentration is associated with CKD progression and its complications [22]. To date, studies regarding leptin in pediatric patients with NS have reported inconsistent results, and its role in this condition remains unclear [23] [24] [25] [26]. Programmed cell death ligand 1 (PD-L1) is a transmembrane protein expressed on immune, epithelial, endothelial cells and various other cell types, and its interaction with PD-1 plays a crucial role in maintaining immune tolerance and regulating immune responses [27]. In malignant diseases, PD-L1 overexpression enables tumor cells to evade cytotoxic T cells, leading to metastasis and poor patient survival [27]. PD-L1 has also been implicated in the pathogenesis of inflammatory diseases, including glomerulonephritis [27] [28]. Experimental studies have shown that PD-L1 has a protective effect in certain kidney diseases, such as ischemic reperfusion-induced acute kidney injury, and nephrotoxic nephritis, both determinant and protective effect in lupus nephritis, but no significant effect in proliferative immune glomerulonephritis [27]. Additionally, increased activation of PD-L1 signaling pathways in glomeruli has been associated with kidney function decline, glomerulosclerosis, and vascular damage in elderly individuals [29]. However, the role of PD-L1 in NS is largely unknown. E-cadherin, a transmembrane adhesion molecule, plays a crucial role in maintaining epithelial barrier integrity and regulating inflammatory signaling pathways in epithelial cells and mononuclear phagocytes [30]. Limited research on E-cadherin in pediatric NS suggests lower urinary levels during the acute phase compared to the remission phase, calling for further investigation into its significance in this patient population [31].

The objective of this study is to examine the parameters of oxidative stress status (TOS, AOPP, PAB, -SH, PON 1 and TAS), inflammatory biomarkers (PTX 3, leptin, PDL-1 and e-cadherin) in pediatric patients with NS during the acute phases of NS in relation to their hypertension status. In addition, we will explore parameter changes and associations across the disease stages.

## Materials and methods

### Subjects and study design

The current prospective study was conducted using a sample of 33 children (22 boys and 11 girls) with idiopathic NS. The median age of the patients was 5 years (interquartile range (IQR) 3–8 years). All patients were diagnosed and treated at the University Children's Hospital in Belgrade, Serbia. The diagnostic criteria for idiopathic NS, treatment protocol, and definition of remission of NS were based on Kidney Disease Improving Global Outcomes (KDIGO) guidelines [32]. After the establishment of the diagnosis of idiopathic NS, patients received standard oral daily prednisolone induction therapy (2 mg/kg, maximum 60mg) for 4 weeks, followed by further lower doses administered on alternate days during remission. None of the patients received lipid-lowering therapy or supplements that affect the anti-oxidant status of patients. We used an online calculator to determine body mass index (BMI) from weight (kg) and height (cm) for children. Blood pressure measurement was measured in a sitting position by using a mercury sphygmomanometer with an appropriate cuff size.

### Biochemical analysis

Blood sampling for this study was performed in patients twice: at the acute phase – the period immediately following the diagnosis of idiopathic NS in all patients (before the initiation of corticosteroid treatment) and during the remission phase – approximately after 4 weeks of prednisolone therapy. The average time between the first and the second point was 40 (IQR 30–50) days. Blood samples were collected into evacuated serum sample tubes after a 12-hour fasting period. The serum was separated by immediate centrifugation at 1500 × g for 10 min at 4°C. Aliquots of each sample were stored at -80°C and thawed immediately before analyses. Serum urea, creatinine, albumin, total protein, total cholesterol (TC), high-density lipoprotein cholesterol (HDL-C) and triglyceride (TG) concentrations were quantified by routine methods on an ILab 300+ analyzer (Instrumentation Laboratory, Milan, Italy). Serum low-density lipoprotein cholesterol (LDL-C) concentration was calculated by the Friedewald formula (LDL-C (mmol/L) = TC - HDL-C - TG/2.2) [33]. A method of reaction with glacial acetic acid and potassium iodide was applied for AOPP measurement [34]. PON1 activity was measured using an ILab 300+ analyzer (Instrumentation Laboratory, Milan, Italy) according to the Richter and Furlong method [35]. Total -SH groups were measured by a colorimetric method using a spectrophotometer [36], whereas a method using 3,3’, 5,5’-tetramethylbenzidine as a chromogen was applied for PAB measurement [14]. Serum TOS and TAS were measured according to the method described by Erel [15] [16]. PTX3 was determined by an enzyme-linked immunosorbent assay (ELISA) commercial kit (Human Pentraxin3 DuoSet ELISA R&D Systems, Minneapolis, USA) according to the recommendations of the manufacturer. The concentration of leptin in serum was determined using an ELISA commercial kit (DRG Instruments, Marburg, Germany). Serum PD-L1 and E-cadherin concentrations were determined by ELISA tests (R&D Systems, Inc. Minneapolis, MN, USA).

### Ethical statement

Informed consent was obtained from children’s caregivers, and the study was carried out under the Helsinki Declaration. The study protocol was approved by the ethics committee of the University of Belgrade Children’s Hospital, Serbia (approval No.13/229) and the Faculty of Pharmacy, University of Belgrade, Serbia (approval No. 25336/2).

### Statistical analysis

Due to a small sample size and the fact that parameters were not normally distributed even after logarithmic transformation, we presented the data using medians and interquartile ranges (IQR). Differences in parameters between the acute and remission phases of NS were assessed using the Wilcoxon signed ranks test. Gender distribution was evaluated using the chi-square test. For comparisons within the acute phase of NS, we employed the Mann–Whitney U test. Correlation analysis was conducted using Spearman’s correlation test. IBM® SPSS® Statistics version 22 software was used for all analyses, and a significance level was set at p < 0.05.

## Results

In Table 1, anthropometric and basic biochemical parameters are presented. The median age of the patients was 5 years (IQR; 3–8 years), and the male-to- female ratio was 22: 11. There were no significant differences in systolic blood pressure (SBP), diastolic blood pressure (DBP), glucose, urea or creatinine between the acute and NS remission phase. Body mass index (BMI) was significantly higher in the acute phase compared with the remission phase of the disease (P=0.001). As expected, the serum total protein and serum albumin levels exhibit a marked decrease in NS when compared with remission phase NS due to proteinuria associated with this condition (P = 0.001 and P < 0.001). In the remission phase, a significant decrease of TC, LDL-C, TG, and an increase in HDL-C concentrations was observed (P < 0.001 for TC, LDL-C and HDL-C; and TG, P = 0.013).

**Table 1:** Clinical data of pediatric patients with nephrotic syndrome (NS) during acute and remission phases. Data are presented as median (interquartile range) and compared using the Wilcoxon signed-rank test. BMI, body mass index; SBP, systolic blood pressure; DBP, diastolic blood pressure; LDL-C, low-density lipoprotein cholesterol; HDL-C, high-density lipoprotein cholesterol; TC, total cholesterol; TG, triglycerides. P < 0.05 was considered statistically significant.

Parameters	NS–acute phase (n=33)	NS–remission phase (n=33)	P-value
Age (years)	5(3–7.5)	5(3–8)	0.068
Gender, M/F	22/11	22/11	1.000
BMI, kg/m^2^	18 (17.3–18.8)	16.5 (15.3–17.7)	0.001
SBP, mmHg	110 (99–120)	103 (100–119)	0.635
DBP, mmHg	70 (60–80)	68 (60–79)	0.422
Glucose, mmol/L	5.1 (4.3–6.1)	4.8 (4.3–5.3)	0.069
Urea, mmol/L	4.3 (3.3–5.9)	4.5 (4.1–5.7)	0.820
Creatinine, μmol/L	34 (26–41)	39 (31–49)	0.086
Total protein, g/L	42 (38–48)	62 (57–68)	0.001
Albumin, g/L	11 (8–18)	31 (26–36)	< 0.001
TC, mmol/L	9.5 (7.1–11.6)	7.1 (6.0–8.3)	< 0.001
LDL-C, mmol/L	7.1 (4.6–8.9)	3.6 (2.8–5.5)	< 0.001
HDL-C, mmol/L	1.3 (0.9–1.7)	2.2 (1.8–2.8)	< 0.001
TG, mmol/L	2.0 (1.3–2.9)	1.7 (1.0–2.0)	0.013

Serum concentrations of oxidative stress and inflammation markers during the acute and remission phases of NS are displayed in Table 2. Significantly higher PON 1 activity and total SH group concentrations were observed in the remission phase of NS compared with the levels in the acute phase (p<0.001 for both variables). The levels of TAS at remission were higher than those in the acute phase but did not reach a significant level (p =0.810). During remission, patients had lower AOPP and PAB levels as compared with in the acute phase, however; it did not reach significant levels (p=0.370). In contrast, TOS levels were slightly increased during remission compared with the acute phase although did not reach a significant level (p=0.290). PTX 3 levels were significantly decreased during remission as compared with its level in acute phase NS (p<0.001). While leptin levels were significantly increased during the remission phase as compared with its levels during the acute phase, PD-L1 and E-cadherin levels were slightly increased (p<0.001, p=0.057, p=0.064; respectively).

**Table 2:** Changes in oxidative stress and inflammatory markers during active and remission phases in children with nephrotic syndrome (NS). Data are presented as median (interquartile range). The comparison was performed by the Wilcoxon signed rank test. AOPP, advanced oxidation protein products; PAB – prooxidant-antioxidant balance; TOS, total oxidant status; PON1, paraoxonase 1; -SH, sulfhydryl groups; TAS, total antioxidant status; PTX 3, pentraxin 3; E-cad, E cadherin; PD-L1, programmed death ligand 1. P < 0.05 was considered statistically significant.

Parameters	Acute phase NS (N=33)	Remission phase NS (N=33)	p-value
TAS, μmol/L	836.74 (752.55–968.12)	857.89 (754.54–1002.53)	0.810
TOS, μmol/L	11.25 (7.83–14.48)	13.30 (9.03–20.65)	0.290
AOPP, μmol/L	51.30 (40.60–67.15)	46.00 (37.33–61.58)	0.168
SH groups, mmol/L	0.234 (0.154–0.365)	0.561 (0.441–0.709)	< 0.001
PON1 activity, U/L	509 (254–994)	578 (341–1140)	< 0.001
PAB, HKU	158.70 (124.30–193.90)	153.70 (131.30–173.43)	0.370
PTX 3, ng/mL	5.451 (3.581–8.348)	3.367 (2.418–5.388)	< 0.001
Leptin, ng/mL	0.407 (0.229–1.377)	2.027 (1.815–2.145)	< 0.001
E-cad, ng/L	622 (520–715)	779 (614–902)	0.064
PDL-1, ng/L	132.50 (85.00–249.17)	280.83 (180.83–462.50)	0.057

As shown in Table 3, to test the association of biochemical parameters, oxidative markers, and inflammation markers with hypertension we divided patients with NS during the acute phase of NS into two groups according to their hypertensive status. Serum AOPP and TOS concentrations were significantly higher in the NS patients with hypertension compared with those without hypertension (p=0.029, p=0.003; respectively). There were no differences between the two groups concerning other investigated parameters.

**Table 3:** Association of hypertension (HT) with BMI, basic biochemical parameters, oxidative stress and inflammation markers in children with nephrotic syndrome in the acute phase. Data are presented as median with interquartile range. The comparison was performed by the Mann–Whitney U test. LDL-C, low-density lipoprotein cholesterol; HDL-C, high-density lipoprotein cholesterol; TC, total cholesterol; TG, triglycerides; AOPP, advanced oxidation protein products; PAB, prooxidant-antioxidant balance; TOS, total oxidant status; PON1, paraoxonase 1; -SH, sulfhydryl groups; TAS, total antioxidant status; PTX 3, pentraxin 3; E-cad, E cadherin; PD-L1, programmed death ligand 1. P < 0.05 was considered statistically significant.

Parameters	Patients with HT (n=8)	Patients without HT (n=25)	P-value
BMI, kg//m^2^	18.6 (18.7–20.1)	17.8 (16.8–18.6)	0.074
Glucose, mmol/L	4.6 (3.7–6.4)	5.33 (4.5–6.1)	0.429
Urea, mmol/L	4.4 (3.3–11.7)	4.0 (3.2–5.5)	0.472
Creatinine, μmol/L	27.5 (24–47)	34.0 (28.3–40.5)	0.420
Total proteins, g/L	42 (37–51)	41 (39–47.8)	0.831
Albumin, g/L	10 (7.5–14.8)	10.5 (7.3–18.5)	0.711
TC, mmol/L	10.7 (7.4–12.5)	9.4 (6.7–11.3)	0.384
LDL-C, mmol/L	7.9(5.0–10.1)	7.0(4.4–8.8)	0.443
HDL-C, mmol/L	1.1 (1.0–1.4)	1.4 (0.9–1.8)	0.119
TG, mmol/L	2.5 (1.5–3.1)	1.8 (1.1–2.8)	0.286
TAS, μmol/L	814.09 (702.07–975.40)	832.35 (745.21–951.70)	0.684
TOS, μmol/L	17.35 (11.68–20.85)	8.70 (7.70–12.00)	0.003
AOPP, μmol/L	67.15 (56.89.90–86.55)	44.60 (36.33–53.93)	0.029
SH, mmol/L	0.31 (0.12–0.50)	0.22 (0.16–0.34)	0.395
PON1, U/L	256.5 (250.5–1352.8)	531.5 (263.5–934.5)	0.760
PAB, HKU	129.4 (122.25–174.15)	162.58 (127.35–297.70)	0.201
PTX 3, ng/mL	5.48 (4.56–10.51)	4.81 (3.17–7.12)	0.241
Leptin, ng/mL	0.29 (0.16–0.58)	0.46 (0.26–1.89)	0.140
E-cad, ng/L	549.00(521.00–634.00)	669.92(518.00–744.00)	0.250
PDL-1, ng/L	129.17(29.17–250.83)	45.84(98.33–262.92)	0.561

**Figure 1:**
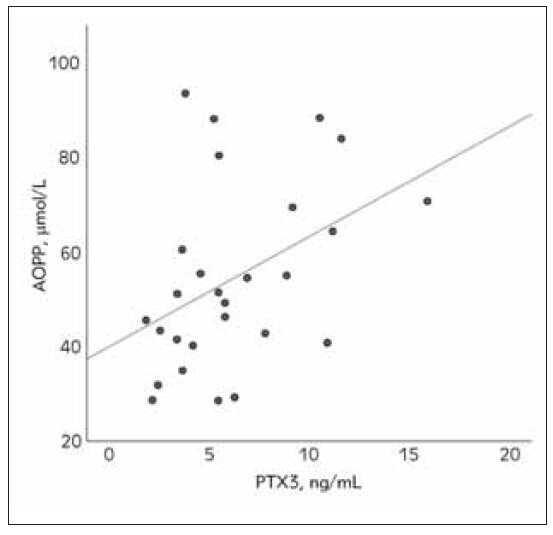
The relationship between advanced oxidation protein products (AOPP) and pentraxin 3 (PTX3) in the acute phase of nephrotic syndrome in pediatric patients (Spearman’s correlation coefficient, rs = 0.42, p=0.027).

**Figure 2:**
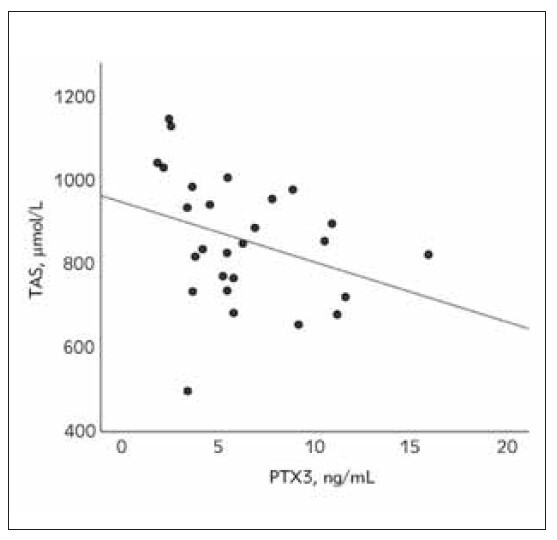
The relationship between total antioxidant status (TAS) and pentraxin 3 (PTX3) in the acute phase of nephrotic syndrome in pediatric patients (Spearman’s correlation coefficient rs=-0.43, p=0.025).

**Figure 3:**
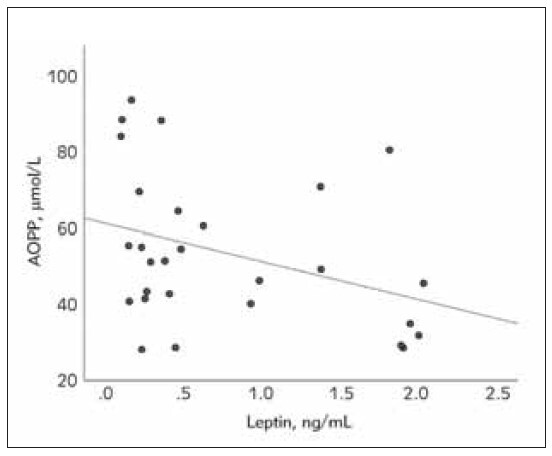
The relationship between total antioxidant status (TAS) and pentraxin 3 (PTX3) in the acute phase of nephrotic syndrome in pediatric patients (Spearman’s correlation coefficient rs=-0.43, p=0.025).

Figure 1, Figure 2, Figure 3 show correlations between oxidative stress and inflammation markers. Serum PTX 3 levels were positively correlated with AOPP and negatively correlated with TAS during acute phase NS (p =0.027, rs =0.42, p=0.025, rs =-0.43; respectively). Serum AOPP levels were inversely correlated with leptin levels during the acute phase of NS (p =0.028, rs =-0.42). The correlations among PTX 3, AOPP, TAS, and leptin were lost during the remission phase of NS.

## Discussion

Oxidative stress, an imbalance between the production of ROS and the body's antioxidant defenses, has been implicated in the etiology of various diseases. The profound impact of oxidative stress on cellular homeostasis and tissue integrity is apparent across various medical conditions, including autoimmune disorders, metabolic diseases, and cardiovascular pathologies [37] [38] [39]. In different medical conditions, like rheumatoid arthritis, diabetes mellitus, atherosclerosis, obesity, and hypertension, the disturbance of redox balance plays an important role in initiating inflammatory responses and worsening disease progression [37] [38]. Furthermore, numerous studies have demonstrated the impact of oxidative stress in pediatric patients with NS [7] [8] [9]. However, the intricate interplay between inflammation, oxidative stress, and hypertension in pediatric NS remains an area that has received limited exploration in the existing literature.

Our study shows that the anti-oxidant capacity of pediatric patients with NS was significantly lower during the acute phase, as demonstrated by lower levels of –SH and PON 1 activity (Table 2). The lower TAS, an indicator of overall anti-oxidant capacity, during the acute phase of NS also supports these findings (Table 2). In line with our results, Karthikeyan et al. [40] have shown that the levels of –SH significantly lower during the acute phase, whereas, in contrast to ours, the level of TAS slightly increased during the acute phase of NS. This discrepancy concerning the level of TAS is most likely due to differences in the assay methods. We used a method that detects potentially all antioxidants present in the plasma including –SH levels, whereas the latter used the method (FRAP, ferric reducing antioxidant power) which does not account for –SH levels [41]. Similar to our results, Bakr et al. [7] have shown that the levels of TAS during the acute phase were lower compared with the remission phase of NS. The authors have also suggested that TAS levels at the acute and remission phases of NS can predict the response to corticosteroid therapy and relapse of the disease. During the acute phase, significant decrease in –SH levels and a slight decrease in TAS levels, likely due to parallel decreases in albumin concentration, the major component of plasma thiols and anti-oxidants. Alternatively, the insignificant change in TAS levels suggests that other components of TAS may not yet be fully restored. In support of this hypothesis, Kniazewska et al. [42] found that the non-enzymatic components of the anti-oxidant system were not fully recovered even in children with long-term remission.

Regarding PON 1 activity, in contrast to our findings, Ece et al. [43] found no difference in PON 1 activity between the acute phase and remission phase of NS. Still, its value was lower compared with patients in steroid-free remission. This discrepancy is most likely because PON 1 activity was measured from two independent patient groups in the latter study. As PON 1 is an HDL particle-associated enzyme, the lower activity of PON 1 during the acute phase in the current study could be the consequence of a lower level of HDL particles, manifested with lower HDL-C concentration (Table 1). It is also possible that a sequel of its consumption is due to increased levels of oxidants.

On the other hand, the slightly higher AOPP and PAB concentrations in the acute phase may be due to the decrease in antioxidant capacity that was observed (Table 2). However, TOS levels which represent the measure of the overall oxidant burden in plasma samples of the patients, tend to increase during the remission phase (Table 2). The slight increase in TOS level in remission is somewhat unexpected, as one might expect this marker to decrease with decreased oxidative stress. However, this increase may reflect ongoing oxidative stress, despite the remission of clinical symptoms. Also, the medications used in the treatment of NS may have oxidative effects that may be responsible for this observation [44]. In line with our study, Fan et al. [9] found no significant difference in the levels of AOPP between the acute and remission phases of NS. The authors have also demonstrated that AOPP is associated with disease severity as their levels were higher in frequently relapsing patients compared with those who are non-relapsing or non-frequently relapsing [9]. Accordingly, we speculate that AOPP can be a marker of a chronic condition, and the insignificant change in AOPP levels between the acute and remission phases of NS is likely to be expected. Overall, the results suggest that there is a shift towards a better oxidative status in the remission phase of NS compared to the acute phase. This may be due to the effect of medications used in the treatment of NS, changes in the immune system, or other factors. However, our results also indicate that there may still be ongoing oxidative stress in the remission phase, which supports the previous study results [7] [9] [10].

Along with oxidative stress, immunological dysregulation and inflammation underpin NS pathogenesis. Previous studies have highlighted elevated proinflammatory cytokines and acute-phase proteins during NS's acute phase [18]. Consistently, our study reveals heightened inflammation, as indicated by elevated pentraxin 3 (PTX 3) levels. To our knowledge, there is no existing literature examining PTX 3 in NS to compare our results directly. However, elevated PTX 3 levels are observed in conditions marked by oxidative stress and inflammation, like acute ischemia-reperfusion kidney injury, acute renal allograft rejection, CVD, and CKD [19] [45] [46]. PTX 3 levels serve as reflective indices of inflammation and tissue injury [21]. While widely acknowledged as a disease biomarker, the involvement of PTX 3 in the pathophysiology of these conditions is a topic of ongoing debate. In ischemia-reperfusion-induced kidney injury, PTX 3 has both protective and detrimental effects [19]. PTX 3 prevents injury by reducing ROS, inhibiting calpain/caspase-3, stabilizing mitochondria and IL-6/Stat3 pathway suppression [47] [48]. Conversely, it might promote injury by inducing adhesion molecules/chemokines, regulating leukocyte recruitment, and overactivating complement in kidney injury models [49] [50]. Elevated PTX 3 levels are associated with higher CKD patient mortality, increased CKD and CVD risk [19] [20] [51]. Endothelial dysfunction, often linked to heightened PTX 3 levels, is hypothesized to contribute to the augmented CVD risk in CKD [52]. Proposed mechanisms elucidate how PTX 3 fosters endothelial dysfunction include, induction of ROS synthesis and inflammation, enhanced uptake of ox-LDL, impaired cholesterol efflux from foam cells, attenuation of cell proliferation and angiogenesis via fibroblast growth factor 2 (FGF2) inactivation, compromised vasorelaxation due to diminished NO synthesis and perturbed signaling pathways [52]. Overall, our results suggest that pentraxin 3 could serve as a potential biomarker for disease activity, aiding in the monitoring and management of patients during different disease stages.

Furthermore, the correlational analysis revealed that PTX3 is significantly associated with AOPP and TAS during the acute phase of NS (Figure 1 & Figure 2). However, these associations were lost during the remission phase, indicating the interplay of oxidative stress and inflammation in the pathophysiology of NS. AOPPs are recognized as markers of oxidative protein damage (mainly albumin), the intensity of oxidative stress, and inflammation [53]. We also found a direct link between PTX 3 and AOPP (Figure 1). Miljkovic et al. [54] have demonstrated that AOPPs are implicated in the interplay of dyslipidemia, oxidative stress, and inflammation, contributing to cardiovascular complications in renal patients. Similarly, several studies have demonstrated that AOPPs could promote atherosclerosis development through increasing oxidative stress, inflammation, modified lipoproteins and inhibition of reverse cholesterol transport [53]. AOPP can induce upregulation of pro-oxidant enzymes and activation of the Wnt/β-catenin signaling pathway in podocytes causing their injury and proteinuria [11]. The suggested mechanisms in the pathogenic role of AOPP in podocyte dysfunction include up-regulation of NADPH oxidase, snail1, canonical transient receptor potential cation channel 6 (TRPC 6), matrix metalloproteinase- 7(MMP-7), activating renin-angiotensin system and inhibition of Wilms tumor protein [11]. In addition, oxidative stress induces the synthesis of PTX3 in other pathological conditions [55] [56]. This might also be the case in NS as AOPP positively correlated with PTX3 during the acute phase of the disease (Figure 2). However, whether the increased level of PTX3 has a protective or detrimental effect on NS requires further study.

PDL1 is an emerging biomarker of inflammation and an immune checkpoint molecule widely studied in cancer-related inflammation [27] [57]. Through its interaction with PD1, it inhibits T-cell activation, and cytokine production, and promotes apoptosis. Malignant cells exploit PD-L1/PD-1 pathways to evade anti-tumor immunity. Elevated PDL1 levels are also noted in non-malignant conditions like cardiovascular diseases and glomerulonephritis [27]. In the context of NS, our investigation revealed elevated PDL1 during its remission phase. This observation finds resonance in recent scholarly endeavors by Guimarães et al. [58] and Tsuji et al. [59], which unearthed analogous trends in the expression of cytotoxic T-lymphocyte-associated protein 4 (CTLA-4), another pivotal immune checkpoint molecule. Besides its interaction with PD-1, a novel facet of PDL1 emerges as it dimerizes with CD80, instigating a cascade that diminishes PD-L1/PD-1 and CTLA- 4/CD80 signaling [60] [61]. This intricate interplay ultimately augments the availability of CD80, a pivotal costimulatory molecule in T-cell activation, thereby favoring the CD80/CD28 signal pathway. The overexpression of CD80 molecules in the acute phase of NS has been well documented [62]. This may inhibit the anti-inflammatory effect of the PD-L1/PD-1 pathway via sequestering PD-L1 as well as activating the inflammatory CD80/CD28 pathway. Hence, we speculate that the change in PD-L1 levels in the current study can be attributed, in part, to the heightened expression of CD80 during the acute phase. Concurrently, glucocorticoids may upregulate PD- 1/PD-L1 pathways [63] [64]. The dynamic modulation in PD-L1 concentration suggests its potential role in shaping immune responses throughout the disease trajectory. The surge in PD-L1 during remission might signify an endeavor to restore immune homeostasis and mitigate inflammation as the disease subsides. This discovery introduces complexity to our comprehension of immune regulation in NS and underscores the intricate interplay between immune checkpoints and disease progression.

Similar to PDL1, E-cadherin has been extensively studied in malignancies [30]. Serving as a cell-cell adhesion molecule, E-cadherin plays a pivotal role in epithelial integrity and signaling [30]. Increased serum levels and tissue expression correlate with malignant progression. Urinary E-cadherin emerges as a specific marker for diabetic kidney injury [65]. Recent findings also link E-cadherin to inflammation and oxidative stress in chronic hepatitis B virus [66]. Regarding NS, limited research exists, with a single study revealing lower urinary E-cadherin in the acute phase versus remission [31]. In line with the previous results, we found lower serum E-cadherin in the acute phase of NS compared with remission, further highlighting its potential significance in the pathophysiology of NS. To understand the exact role of E-cadherin in NS warrants further in-depth analysis.

Leptin, an adipocyte-derived cytokine (adipokine), has been thought to link obesity related-inflammation with cardiovascular disease and kidney injury. It has also been proposed that leptin may play a very important role in the pathophysiology of NS [67]. Dinleyic et al. [67] have demonstrated the association of low serum levels of leptin with proteinuria, hypoproteinemia, and hyperlipidemia. However, studies have reported conflicting results regarding the level of serum leptin in the acute phase of NS compared with the remission phase levels. Some studies found lower [25] [26] [67], and others found unchanged [23] [68] or higher [69] levels of leptin in the acute phase of NS compared with the remission phase. Our results are consistent with those studies that found lower levels of leptin during the acute phase of NS. Previous studies have proposed that low levels of leptin resulted from increased urinary excretion. Interestingly, we found a negative and significant association between AOPP and leptin during the acute phase (Figure 3). This suggested that, alongside with increased urinary excretion, increased oxidative stress may contribute to the downregulation of leptin synthesis. Also, the absence of correlation between serum leptin levels and BMI in acute phase NS, but the retainment of significant positive correlation during the remission phase suggests that leptin production and adipose tissue were affected in NS (data not shown).

Hypertension is frequently observed complication of NS, in particular during the acute phase of the disease [3] [4]. Our results show there were noticeable distinctions between pediatric patients with and without hypertension concerning parameters of oxidative stress during the acute phase of NS (Table 3). Those with hypertension displayed higher levels of AOPP and TOS, indicating an increase in oxidative stress in pediatric patients with NS and hypertension. To our knowledge, this is the first study about the relationship between hypertension and oxidative stress in the context of NS. However, our results align with studies that link hypertension to increased levels of oxidative stress in various disease conditions [6] [37] [70] [71]. It is worth mentioning that inflammatory markers such as PTX3, leptin, PDL1, and E-cadherin did not exhibit differences between the two groups during this phase. The lack of significant changes in inflammatory markers during the acute phase contradicts some reports associating hypertension with inflammation [5] [37]. This discrepancy may be due to the specific context of nephrotic syndrome, which could have unique inflammatory pathways.

A relatively small patient population is one of the limitations of this study, which makes it especially difficult to interpret subgroup findings. Another limitation of our study is that inflammatory and oxidative status markers were not assessed in the glucocorticoid- off remission phase. Investigations should be continued with a larger study group and for a longer time of observation.

### Conclusions and future directions

The results of our study demonstrate that children with NS experience an intricate interplay between oxidative stress, inflammation, and hypertension. It is crucial to acknowledge that NS patients with hypertension have pronounced oxidative stress (higher TOS and AOPP levels) during the acute phase of NS. This novel correlation unveils a previously unrecognized connection between hypertension and oxidative stress in the context of pediatric NS. Additionally, there is a noticeable reduction in antioxidant capacity during the acute phase, as demonstrated by diminished levels of -SH, PON 1, and TAS. This decline, which was also confirmed by previous studies, implies that oxidative stress may contribute to the onset and progression of NS.

While certain markers of inflammation exhibit no notable differences, indicating distinct inflammatory pathways in NS, PTX 3 emerges as a promising biomarker for disease activity. PTX 3 is correlated with oxidative stress markers specifically during the acute phase of NS, demonstrating a dynamic interaction between inflammation and oxidative stress in this renal disorder. These findings contribute to a more nuanced understanding of the underlying mechanisms driving NS pathophysiology. Furthermore, our study brings attention to the potential involvement of leptin, establishing a connection to oxidative stress. This new association highlights the multifaceted nature of the factors influencing NS progression and opens up avenues for further investigation into the role of leptin in the context of oxidative stress.

Given these observations, our study offers valuable insights into the intricate connection between oxidative stress, inflammation, and hypertension in pediatric nephrotic syndrome. Moving forward, our results provide a solid experimental foundation for future research and the development of antioxidant medications aimed at reducing oxidative stress in the management of pediatric NS and its complications. Based on the available data, it is evident that pediatric patients with NS have close associations with alterations in redox status and steroid therapy may not be adequate to alleviate oxidative stress in patients with NS fully. It is widely recognized that oxidative stress contributes to the subsequent development of cardiovascular complications in high-risk populations. As a result, addressing oxidative stress is an extremely important aspect of disease prevention and management. Consequently, in addition to the typical steroid therapy, changes in lifestyle (such as diets high in antioxidants and regular physical activity), as well as targeted therapeutic interventions, are considered potential strategies to reduce the negative effects of oxidative stress. Leptin functions as a molecular connection between obesity-induced oxidative stress and its complications such as insulin resistance, type 2 diabetes, metabolic syndrome, liver fibrosis, and cardiovascular diseases [37] [38] [72]. Conversely, in the present study, the inverse relationship between leptin and oxidative stress indicates its protective effect in pediatric nephrotic syndrome. Thus, future studies should explore the potential therapeutic targeting of the leptin-oxidative stress loop in the pediatric population with NS.

## Dodatak

### Acknowledgments

This work was supported by the Ministry of Science, Technological Development and Innovation, Republic of Serbia (Grant Agreement with the University of Belgrade, Faculty of Pharmacy No: 451-03-47/2023-01/200161).

### Conflict of interest statement

All the authors declare that they have no conflict of interest in this work.

### List of abbreviations

AOPP, advanced oxidation protein products;<br>BMI, body mass index;<br>DBP, diastolic blood pressure;<br>ELISA, enzyme-linked immunosorbent assay;<br>HDL-C, high-density lipoprotein cholesterol;<br>LDLC, low-density lipoprotein cholesterol;<br>total antioxidant status;<br>NS, nephrotic syndrome;<br>PAB, prooxidant-antioxidant balance;<br>-SH, sulfhydryl group;<br>SBP, systolic blood pressure;<br>PON 1, paraoxonase;<br>PTX 3, pentraxin 3;<br>PD-L1, programmed cell death ligand 1;<br>TC, total cholesterol;<br>TG, triglyceride;<br>TAS, total antioxidant status;<br>TOS, total oxidation status
